# A Pulse Wave Velocity Based Method to Assess the Mean Arterial Blood Pressure Limits of Autoregulation in Peripheral Arteries

**DOI:** 10.3389/fphys.2017.00855

**Published:** 2017-11-02

**Authors:** Ananya Tripathi, Yurie Obata, Pavel Ruzankin, Narwan Askaryar, Dan E. Berkowitz, Jochen Steppan, Viachaslau Barodka

**Affiliations:** ^1^Department of Anesthesiology and Critical Care Medicine, Johns Hopkins University School of Medicine, Baltimore, MD, United States; ^2^Sobolev Institute of Mathematics, Novosibirsk, Russia; ^3^Department of Mathematics and Mechanics, Novosibirsk State University, Novosibirsk, Russia

**Keywords:** limits of autoregulation, pulse wave velocity, pulse arrival time, hydrostatic pressure, peripheral artery

## Abstract

**Background:** Constant blood flow despite changes in blood pressure, a phenomenon called autoregulation, has been demonstrated for various organ systems. We hypothesized that by changing hydrostatic pressures in peripheral arteries, we can establish these limits of autoregulation in peripheral arteries based on local pulse wave velocity (PWV).

**Methods:** Electrocardiogram and plethysmograph waveforms were recorded at the left and right index fingers in 18 healthy volunteers. Each subject changed their left arm position, keeping the right arm stationary. Pulse arrival times (PAT) at both fingers were measured and used to calculate PWV. We calculated ΔPAT (ΔPWV), the differences between the left and right PATs (PWVs), and compared them to the respective calculated blood pressure at the left index fingertip to derive the limits of autoregulation.

**Results**: ΔPAT decreased and ΔPWV increased exponentially at low blood pressures in the fingertip up to a blood pressure of 70 mmHg, after which changes in ΔPAT and ΔPWV were minimal. The empirically chosen 20 mmHg window (75–95 mmHg) was confirmed to be within the autoregulatory limit (slope = 0.097, *p* = 0.56). ΔPAT and ΔPWV within a 20 mmHg moving window were not significantly different from the respective data points within the control 75–95 mmHg window when the pressure at the fingertip was between 56 and 110 mmHg for ΔPAT and between 57 and 112 mmHg for ΔPWV.

**Conclusions**: Changes in hydrostatic pressure due to changes in arm position significantly affect peripheral arterial stiffness as assessed by ΔPAT and ΔPWV, allowing us to estimate peripheral autoregulation limits based on PWV.

## Introduction

Various organs are equipped with an autoregulation mechanism in order to maintain constant blood flow as local blood pressure changes (Peterson et al., 2011). These autoregulation mechanisms rely on robust arterial reactivity: as blood pressure decreases, muscular arterioles dilate, and as blood pressure increases, muscular arterioles constrict (Meng and Gelb, 2015). The lower limit of autoregulation represents the pressure at which arteries are maximally dilated and organ perfusion becomes pressure dependent. If blood pressure falls below the lower limit of autoregulation, then blood flow decreases. The upper limit of autoregulation represents the pressure at which the arteries are maximally constricted (Meng and Gelb, 2015). If blood pressure goes above the upper limit of autoregulation, then blood flow increases. This physiological mechanism has been demonstrated for the brain, kidney, bone, intestinal tracts, and even the peripheral arteries in the lower extremities (Lassen, 1959; Johnson, 1967; Cupples and Braam, 2007; Vogt et al., 2013).

Limits of autoregulation have been assessed by measuring arterial blood flow velocity during changes of mean arterial blood pressure (Czosnyka et al., 2009). However, it remains challenging to measure the lower limit of autoregulation in humans, especially in vital organs, as this requires inducing potentially dangerously low mean arterial blood pressures, which of course is not ethically acceptable. However, peripheral arteries are routinely exposed to periods of low blood pressures. Raising the arm with respect to the level of the heart is a non-invasive way to decrease local blood pressure by introducing a hydrostatic pressure gradient. Decreasing local blood pressure then leads to a physiologic decrease in wall tension of the corresponding peripheral arteriole. The resultant changes in wall tension elicited by hydrostatic pressure gradients can be quantified precisely since blood density is known and arm length can be measured easily (Butlin et al., 2015).

Measuring arterial blood flow velocity to detect the limits of autoregulation can be done in real time and non-invasively using Doppler ultrasound, a technique which is operator-dependent and time consuming. It has been reported previously that pulse wave velocity (PWV) and the velocity of flow velocity wave transmission are nearly identical (Pai and Shah, 1999). Hence as blood pressure decreases, PWV should have a similar relationship to flow and exhibit a noticeable lower limit of autoregulation.

PWV is a measure of arterial stiffness and depends on the elasticity of the arterial wall (Bramwell and Hill, 1922). The elasticity of the arterial wall in turn depends on both the intrinsic arterial wall composition and wall tension. We have shown previously that PWV increases as MAP increases (Steppan et al., 2014). However, the relationship between PWV and MAP is not linear as PWV increases exponentially at high blood pressures, whereas the changes in PWV are relatively minor when blood pressure is in the normal range. This phenomenon is based on the composition and structure of the arterial wall and its load-bearing components (mainly elastin and collagen) (Steppan et al., 2014). Similar to active blood flow autoregulation, the elastin fibers dampen the changes in blood pressure, thereby maintaining a relatively constant PWV.

We hypothesize that by inducing hydrostatic pressure changes in peripheral arteries by changing arm position, we can evaluate the limits of autoregulation in those arteries via assessing changes in local PWV.

## Methods and materials

### Subjects

This study was approved by the Johns Hopkins Medicine Institutional Review Board (IRB00074229). 18 healthy volunteers were recruited to participate through email or word of mouth following Institutional Review Board approval. Informed oral consent was obtained from all subjects. Inclusion criteria were: healthy adults, aged 18–50 years, and both genders. Exclusion criteria were: subject refusal to participate, known cardiovascular disease of any kind, and pregnancy. After confirming that each subject could participate in the study, each subject self-reported her/his weight, height, age, handedness, and gender.

### Study protocol

A standard 3 lead electrocardiogram (ECG; Bio Amp FE132, ADInstruments, Australia) was placed on the volunteer to allow for continuous measurement of electrical cardiac activity. We utilized clinically used standard lead locations as suggested by the American Heart Association (AHA) Scientific Statement on Practice Standards for Electrocardiographic Monitoring in Hospital Settings (Drew et al., 2004). A PowerLab analog to digital converter (PowerLab 4/26, ADInstruments, Australia) along with LabChart 8.0 software (LabChart8, Ad Instruments Ltd, Australia) were both used to convert and digitally record the data. Capillary plethysmograph sensors (MLT1020PPG IR Plethysmograph, ADInstruments, Australia) were then placed on the left and right index fingers. The ECG and plethysmograph simultaneously recorded data for each position for 60 s per position. The subject was seated on a chair with both arm rests at equal heights at the level of the heart. The first position (position A0) was used for a baseline measurement in which the subject had her/his arms resting on the armrests in a horizontal position, at the same height as the subject's heart. The next position (position A1) was identical to position A0 with the difference being that the volunteers left forearm was raised upward, such that subject's forearm was perpendicular to the subject's upper-arm, which was resting on the armrest. The subject was then instructed to switch to the up position (position A2), in which the subject raised her/his left arm vertically up above their head while keeping her/his right arm in the same position as it was resting before. The subject then went back to the horizontal position (position B0), with both arms were resting along the same plane as the heart as in position A0. The subject then took her/his left forearm vertically downward, in a half-down position, while keeping her/his upper-arm flat on the armrest (position B1), such as in position A1, just in the opposite direction. The subject did this while holding the right arm in the same position as before. The subject then switched to the final down position (position B2) in which he/she extended her/his left arm all the way down while keeping her/his right arm in the same position as it was resting before (opposite to position A2). After position B2 was completed, the sensors were removed and the subject's blood pressure was recorded using the oscillometric method over the brachial artery. Several lengths were then measured: wingspan (distance from left index finger to right index finger with both arms in 90° lateral extension), half wingspan on the left side (distance from sternal notch to left index finger with left arm in 90° lateral extension), the distance between the sternal notch and the axilla on the left arm, the distance between the axilla and the elbow on the left arm, the distance between the elbow and the wrist on the left arm, and the distance between the wrist and the left index finger tip on the left arm.

### Data extraction

Using the collected data, the pulse arrival time (PAT) to both the left and the right index fingertip was calculated by automated algorithm as the time delay between each R-wave peak on the ECG waveform and the first positive inflection on the plethysmograph tracing for both the left and the right side.

### Calculations

PWV for both the left (Equation 1) and right (Equation 2) sides were determined using the measured hemi-span (sternal notch to tip of the index finger) lengths divided by the PAT.

(1) $$\begin{array}{r} PWV_{Left}=\frac{d_{wingspan}}{2PAT_{Left}} \end{array}$$

(2) $$\begin{array}{r} PWV_{Right}=\frac{d_{wingspan}}{2PAT_{Right}} \end{array}$$

We also calculated the difference in PAT values (ΔPAT) between the left and right side and the difference in PWV (ΔPWV) between the left and right side at each position keeping right arm at the level of the heart (Equations 3 and 4).

(3) $$\begin{array}{r} \Delta PAT\;=\;PAT_{Left}-PAT_{Right} \end{array}$$

(4) $$\begin{array}{r} \Delta PWV\;=\;PWV_{Left}-PWV_{Right} \end{array}$$

The hydrostatic blood pressure changes were quantified by multiplying the density of blood (ρ = 1,060 kg/m3), gravity related acceleration (*g* = 9.81 m/s^2^), and the height of the fluid column *(d)* (Equation 5). To convert the hydrostatic pressure from Pascals to mmHg, the obtained value was multiplied by a conversion factor of 0.0075 (Butlin et al., 2015).

(5) $$\begin{array}{r} Hydrostatic\;pressure\;=\;0.0075\rho gd \end{array}$$

For our analysis, we quantified the maximum pressure change at the tip of the finger. Hydrostatic pressures calculated for the up positions (A1 and A2) were defined as negative; those calculated for the down positions (B1 and B2) were positive. To calculate the total blood pressure (accounting for both systemic and hydrostatic pressure), the mean arterial pressure (MAP), defined by Equation (6), was added to the hydrostatic pressure (Equation 7).

(6) $$\begin{array}{r} MAP\;=\;DBP\;+\;\left(\left[SBP-DBP\right]/3\right) \end{array}$$

(7) $$\begin{array}{r} Calculated\;pressure\;=\;Hydrostatic\;pressure\;+\;MAP \end{array}$$

### Statistical analysis

Based on the results of the previous study, we estimated that a sample of 3 subjects would provide adequate power [80% power for a mean (SD) difference of 42.7 ms (11.0 ms)] for the change in PAT between horizontal and up arm position at an alpha level of 0.05 in a two-sided paired *t*-test (Foo et al., 2005). Based on the results of our previous study, we estimated that a sample of 18 subjects would provide adequate power [80% power for a mean (SD) difference of 8.78 ms (12.40 ms)] for the change in PAT between horizontal and down arm position at an alpha level of 0.05 in a two-sided paired *t*-test (Obata et al., 2017).

Paired *t*-tests were used to compare the PATs for each arm between the initial horizontal position (position A0) and the horizontal position after arm raise (position B0) and to compare the PAT and PWV values measured at different left arm positions to the respective values of the right arm, which was constantly kept at the level of the heart. A repeated measure one-way analysis of variance (ANOVA) test was used with the Dunnett's multiple comparisons test to check for differences between PAT, ΔPAT, PWV and ΔPWV at each position. We performed a non-linear regression model to assess the relationship between hydrostatic blood pressure change at the tip of the finger and the resultant change in PAT between left and right arm (ΔPAT), the relationship between the calculated blood pressure at the tip of the finger and the ΔPAT, and the relationship between the calculated blood pressure at the tip of the finger and ΔPWV. These analyses were performed with GraphPad Prism version 6.0 (GraphPad Software, San Diego, California, USA). All tests were two-sided. A *p*-value less than 0.05 was considered to be statistically significant.

To detect the point of inflection of ΔPAT or ΔPWV vs. calculated pressure at the left fingertip relationship, we first created a smoothed curve by applying a LOESS (locally weighted scatter-plot smoother)-smoothed filter to all observed data points. The inflection point of the curve is the point which is maximally distant from the line connecting the curve's ends (Lepeschkin and Surawicz, 1953). The method is robust with respect to the observations' perturbations and is invariant with respect to scaling of the axes. We used the standard LOESS function from the R package (R foundation for Statistical Computing, Vienna, Austria) with values of the span parameter 0.2, 0.4, and 0.75.

To detect the limits of autoregulation we compared the distribution of ΔPAT and ΔPWV observations in ±10 mmHg moving windows to the respective distribution within a known autoregulation range and to the right of the inflection point by the two-sided Mann-Whitney test. To confirm that the empirically chosen control range of calculated blood pressure is in fact within the limits of autoregulation, we calculated the linear regression slope and checked to see if the slope was statistically different from 0. Slopes not different from 0 indicate that the data points within control range are within the autoregulation limit as they show consistent PWV values despite changes in blood pressure. The relationship between the *p*-values of a Mann-Whitney test and blood pressure at the fingertip was plotted. The pressure values at which the plots cross the *p*-value of 0.05 (5% level) were considered to be the lower and higher limits of autoregulation.

## Results

Demographic characteristics and blood pressure values are summarized in Table 1. Each position's average PAT of the left and right arm, ΔPAT, and PWV is summarized in Table 2. The mean age of the volunteers was approximately 32 years and ranged from 18 to 42 years. At rest, the average systolic blood pressure (SBP) was 115.3 ± 24.5 mmHg and the average diastolic blood pressure (DBP) was 68.6 ± 14.5 mmHg.

**Table 1:** Cohort demographics summary (Mean ± SD).

	**Mean ± SD (*n* = 18)**
Age [years]	32.2 ± 6.9
Weight [kg]	66.7 ± 14.0
Height [cm]	169.9 ± 6.9
SBP [mmHg]	115.3 ± 24.5
DBP [mmHg]	68.6 ± 14.5
MAP [mmHg]	84.1 ± 17.2
Wingspan [cm]	170.7 ± 8.4
Half wingspan [cm]	85.3 ± 4.2
d _sternalnotch−fingertip_ [left; cm]	85.3 ± 4.2
d _sternalnotch−axilla_ [left; cm]	20.1 ± 1.7
d _axilla−elbow_ [left; cm]	22.8 ± 2.0
d _elbow−wrist_ [left; cm]	24.3 ± 2.0
d _wrist−fingertip_ [left; cm]	18.1 ± 1.8

*SD, Standard deviation; SBP, Systolic blood pressure; DBP, Diastolic blood pressure; MAP, Mean arterial pressure*.

**Table 2:** Measured and calculated data summary (Mean ± SD).

	**A0**	**A1**	**A2**	**B0**	**B1**	**B2**
PAT_Left_ [ms]	218.6 ± 24.2	229.5 ± 26.4	264.8 ± 37.6	220.0 ± 21.1	215.5 ± 22.7	209.4 ± 22.6
PAT_Right_ [ms]	214.8 ± 24.1	215.6 ± 24.8	211.5 ± 24.3	212.7 ± 23.5	213.2 ± 24.4	212.2 ± 24.1
ΔPAT [ms]	3.8 ± 9.1	14.1 ± 12.4	53.8 ± 22.8	7.2 ± 6.8	2.4 ± 7.2	−1.9 ± 9.5
PWV_Left_ [m/s]	3.9 ± 0.4	3.8 ± 0.5	3.3 ± 0.5	3.9 ± 0.3	4.0 ± 0.4	4.1 ± 0.4
PWV_Right_ [m/s]	4.0 ± 0.4	4.0 ± 0.4	4.1 ± 0.4	4.0 ± 0.4	4.0 ± 0.4	4.1 ± 0.4
ΔPWV [m/s]	−0.1 ± 0.2	−0.2 ± 0.2	−0.8 ± 0.3	−0.1 ± 0.1	0.0 ± 0.1	0.0 ± 0.1

*SD, Standard deviation; PAT_Left_, pulse arrival time at the left index finger; PAT_Right_, pulse arrival time at the right index finger; ΔPAT, difference between the pulse arrival time at the left index finger and right index finger; PWV_Left_, pulse wave velocity at the left index finger; PWV_Right_, pulse wave velocity at the right index finger; position A0, baseline horizontal control position; position A1, left forearm extended vertically toward ceiling (half-up); position A2. left forearm and upper-arm extended vertically toward ceiling (up); position B0, horizontal position in the middle of the trial; position B1, left forearm extended vertically toward floor (half-down); position B2, left forearm and upper-arm extended vertically toward floor (down); ms, milliseconds; m/s, meters per second*.

### PAT analysis

The PAT at positions A0 and B0 were compared using a *t*-test to determine if they were significantly different between the two horizontal readings. The average left PAT values at position A0 were not significantly different from the average left PAT values at position B0 (*p* = 0.508); similarly, the average right PAT at position A0 was not significantly different from the average right PATs at position B0 (*p* = 0.064).

The left PATs for each position were compared to their respective horizontal right PATs (Supplementary Figure 1). Left-sided PATs were significantly higher than right-sided PATs at position A1 (*p* = 0.0002), position A2 (*p* < 0.0001), and position B0 (*p* = 0.0003).

Left PATs at each position were compared to the left PAT at position A0 (Supplementary Figure 2A). Relative to the left PAT at position A0, the left PAT values were significantly higher at position A1 (*p* = 0.005) and position A2 (*p* = 0.0001) and significantly lower at position B2 (*p* = 0.003). The right PAT values were compared to the right PATs at position A0 (Supplementary Figure 2B). The right PATs were significantly higher at position A2 than those at position A0 (*p* = 0.023), despite only minimal changes. ΔPATs at each position were compared to the ΔPAT values at position A0 (Supplementary Figure 2C). Relative to the ΔPAT at position A0, the ΔPAT values were significantly higher at position A1 (*p* = 0.005) and position A2 (*p* = 0.0001).

### PWV analysis

Left-sided PWV values for each position were compared to their respective horizontal right-sided PWV values (Supplementary Figure 3). Left-sided PWVs were significantly lower than the right-sided values at position A1 (*p* < 0.0001), position A2 (*p* < 0.0001), and position B0 (*p* = 0.0003).

PWV values on the left were compared to the left PWV values at position A0 (Supplementary Figure 4A). The left side PWV values at position A0 were significantly higher than the left side PWV values at position A1 (*p* = 0.002) and position A2 (*p* = 0.0001). The left side PWV values at position A0 were significantly lower than the left side PWV values at position B2 (*p* = 0.002). Right side PWV values were compared to right side PWV values at position A0 (Supplementary Figure 4B). The right values at position A0 were significantly higher than those at position A2 (*p* = 0.011). ΔPWV values were compared to ΔPWVs at position A0 (Supplementary Figure 4C). Relative to those at position A0, ΔPWV was significantly lower at position A1 (*p* = 0.004) and position A2 (*p* = 0.0001).

### Pressure analysis

**Figure 1:**
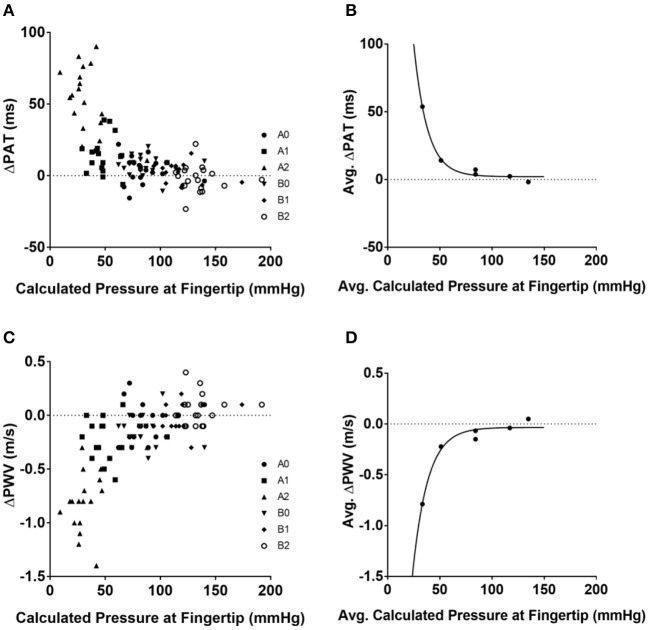
Relationships between blood pressure at the tip of finger and ΔPAT and ΔPWV. Calculated pressure values compared to ΔPAT and ΔPWV values. **(A)** Calculated blood pressure at the tip of finger for each individual subject at each position and ΔPAT. **(B)** Average blood pressure at the tip of finger and average ΔPAT values with the generated non-linear regression model. **(C)** Calculated blood pressure at the tip of finger for each individual subject at each position and ΔPWV values. **(D)** Average blood pressure at the tip of finger and average ΔPWV values with the generated non-linear regression model. ΔPAT, difference between the pulse arrival time at the left index finger and right index finger; ΔPWV, difference between the pulse wave velocity at the left index finger and right index finger; ms, milliseconds; m/s, meters per second.

The hydrostatic pressure was calculated using (Equation 5) (Table 3). The convention implemented for hydrostatic pressure was such that if the left arm was either in position A1 or A2—that is, half-up or fully up—then the hydrostatic pressures were considered to be negative; conversely, if the left arm was in position B1 or B2, in the half-down or complete down position, then the hydrostatic pressures were considered to be positive. The pressure at the fingertip for each position was calculated using Equation (6) (Table 3) and the resulting values were plotted against ΔPAT (Figure 1A). The average pressure values at the fingertips were then calculated and plotted against the average ΔPAT values (Figure 1B). A non-linear one-phase regression model was generated for the average pressure at the fingertips vs. ΔPAT, yielding (Equation 8). The regression model had a coefficient of determination of 0.98, indicating a strong fit for the data.

(8) $$\begin{array}{r} \Delta PAT\;=\;6.78\;\times \;10^{2}\;\times \;e^{\left(-0.08\times \;pressure\;at\;the\;fingertips\right)}\;+\;2.14 \end{array}$$

**Table 3:** Pressure data summary (Mean ± SD).

**Position**	**Hydrostatic pressure [mmHg]**	**Actual pressure [mmHg]**
A0	0.0 ± 0.0	84.1 ± 17.2
A1	−33.1 ± 2.0	51.0 ± 17.2
A2	−50.8 ± 2.9	33.3 ± 16.9
B0	0.0 ± 0.0	84.1 ± 17.2
B1	33.1 ± 2.9	117.2 ± 17.5
B2	50.8 ± 2.9	134.9 ± 18.0

*SD, Standard deviation; Position A0, baseline horizontal control position; position A1, left forearm extended vertically toward ceiling (half-up); position A2, left forearm and upper-arm extended vertically toward ceiling (up); position B0, horizontal position in the middle of the trial; position B1, left forearm extended vertically toward floor (half-down); position B2, left forearm and upper-arm extended vertically toward floor (down)*.

The ΔPWV values were also compared to the calculated pressure at the fingertips (Figure 1C); the average ΔPWVs were plotted against the average pressure at the fingertips at their respective positions (Figure 1D). A non-linear one-phase regression model was generated, yielding Equation (9). This regression model had a coefficient of determination of 0.96.

(9) $$\begin{array}{r} \Delta PWV\;=\;-\;7.58\;\times \;e^{\left(-0.07\times \;pressure\;at\;the\;fingertips\right)}\;-\;0.03 \end{array}$$

### Estimation of the inflection point

**Figure 2:**
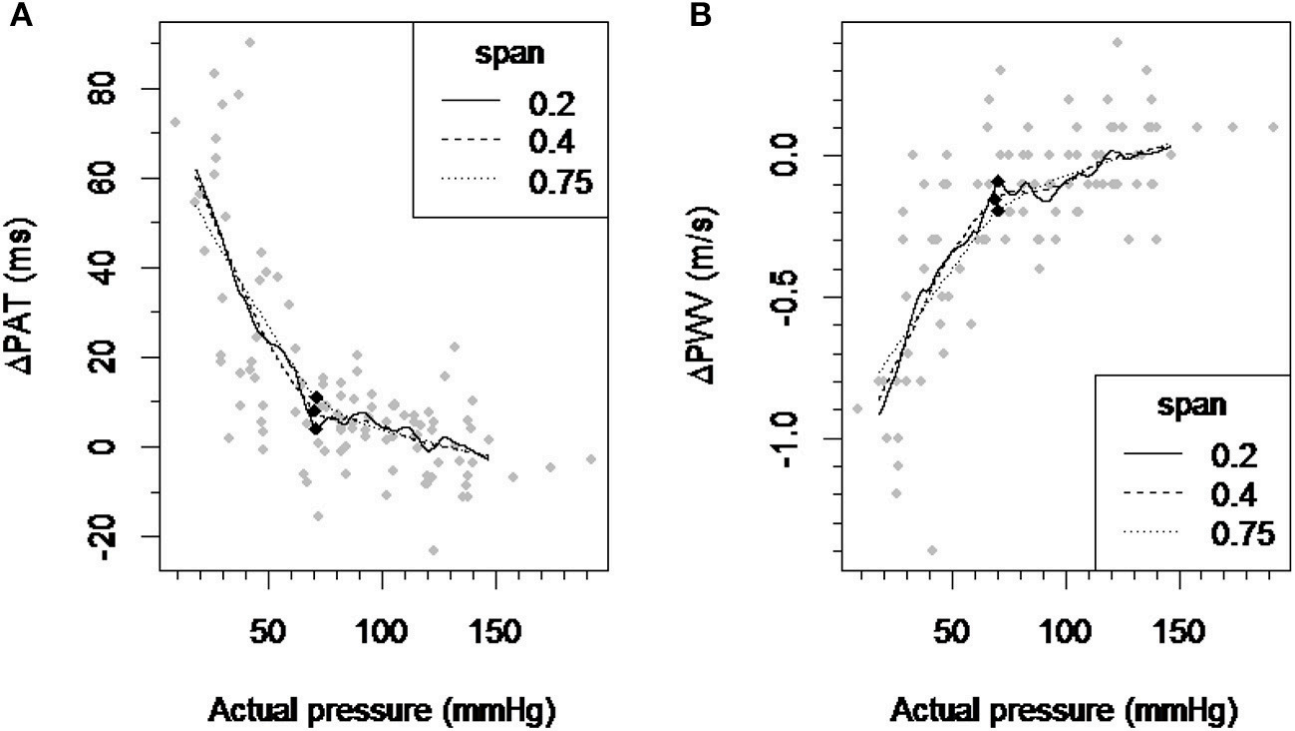
Detection of the inflection point of the ΔPAT and ΔPWV over pressure relationship. **(A)** A scatter plot of ΔPAT vs. pressure and smoothed curves by applying the LOESS-smoothed filter to all observations with the span parameter values of 0.2, 0.4, and 0.75. **(B)** A scatter plot of ΔPWV vs. pressure and smoothed curves by applying the LOESS-smoothed filter to all observations with the span parameter values of 0.2, 0.4, and 0.75. Each black square indicates the inflection point which maximally distant from the line connecting the curve's ends. ΔPAT, difference between the pulse arrival time at the left index finger and right index finger; ΔPWV, difference between the pulse wave velocity at the left index finger and right index finger; ms, milliseconds; m/s, meters per second.

The inflection point for ΔPAT over pressure relationship was detected at 70.67, 70.26, and 71.36 mmHg for the LOESS function span parameter of 0.2, 0.4, and 0.75 respectively (Figure 2A). The inflection point for ΔPWV over the pressure relationship was detected at 70.55, 69.02, and 70.61 mmHg for the LOESS function span parameter of 0.2, 0.4, and 0.75 respectively (Figure 2B). This means that the window with the lowest regression slope within the autoregulation limit will be located at pressures above 70–71 mmHg.

### Estimation of the control blood pressure window within the limits of autoregulation

We chose a ±10 mmHg window to the right of the inflection point, empirically choosing 75–95 mmHg. The estimated regression slope for all data points within the 75–95 mmHg window was 0.097 with *p* = 0.69, indicating that it is not statistically different from the regression slope 0 and the 75–95 mmHg window is in fact within limits of autoregulation. We chose a second window of 80–100 mmHg, with the estimated regression slope for all data points within this window being 0.25 with *p* = 0.36, indicating that it is not statistically different from a regression slope 0 and the 80–100 mmHg window is within the limits of autoregulation.

### Estimation of the limits of regional autoregulation

**Figure 3:**
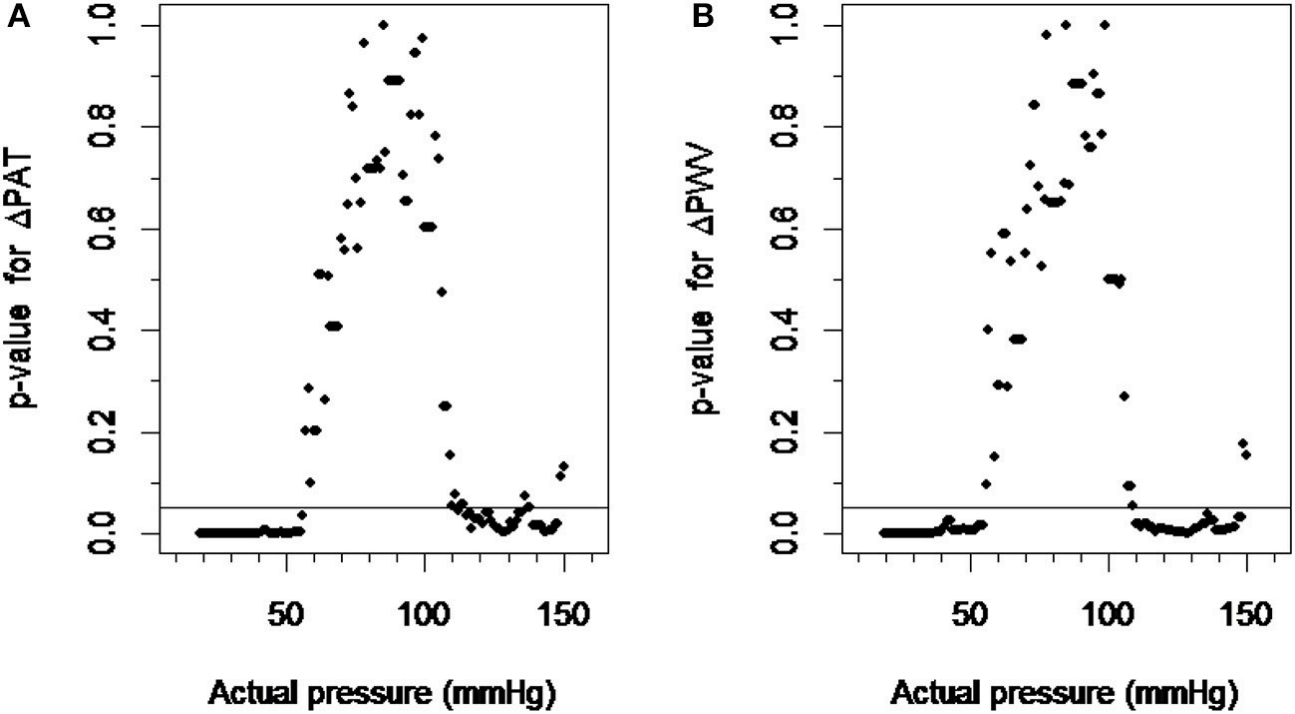
Detection of the limits of autoregulation. **(A)** The scatter plot between *p*-values derived from ΔPAT over pressure relationship and actual pressure. **(B)** The scatter plot between *p*-values derived from ΔPWV over pressure relationship and actual pressure. The pressure values at which the plots cross the *p*-value of 0.05 were considered the lower and higher limits of autoregulation. ΔPAT, difference between the pulse arrival time at the left index finger and right index finger; ΔPWV, difference between the pulse wave velocity at the left index finger and right index finger.

The ΔPAT data within a ±10 mmHg moving window compared to the ΔPAT data within a control window ranging from 75 to 95 mmHg was significant when blood pressures were below 56 mmHg or above 110 mmHg (Figure 3A). Similarly the ΔPWV data within ±10 mmHg moving window compared to the ΔPWV data within a control window ranging from 75 to 95 mmHg was significant when blood pressure was below 57 mmHg and above 112 mmHg. (Figure 3B). Changing the control window from 75–95 mmHg to 80–100 mmHg did not change the results. Hence, the lower limit of autoregulation was detected at 56 mmHg with the *p*-value graph derived from ΔPAT over the calculated blood pressure at the tip of finger relationship (Figure 3A) and at 57 mmHg for the *p*-value graph derived from ΔPWV over blood pressure at the tip of finger relationship (Figure 3B). The upper limit of autoregulation was detected at 110 mmHg on the *p*-value graph derived from ΔPAT over the pressure relationship (Figure 3A) and at 112 mmHg on the *p*-value graph derived from ΔPWV over the pressure relationship (Figure 3B).

## Discussion

In our study, we confirmed that changes in hydrostatic pressure due to changes in arm position significantly affect peripheral PWV and PAT in healthy subjects. In addition, our data suggests the existence of autoregulation in the peripheral arteries of the arm. The average calculated MAP in the study subjects was 84 mmHg. The average length of the arm was 85 cm, which corresponds to an average 66 mmHg decrease in pressure due to hydrostatic effects when the arm is up. Hence, the effective distending pressure at the tip of the fingers is 18 mmHg (84 mmHg – 66 mmHg) in the arm up position and 150 mmHg (84 mmHg + 66 mmHg) in arm down position. As blood pressure at the tip of finger drops below 56 mmHg, we found sudden changes in both ΔPAT or ΔPWV compared to ΔPAT or ΔPWV at a known autoregulation range of 75–95 mmHg, which is consistent with the theory that the vasculature operates on distending pressures below the lower limit of autoregulation. We chose a control distending blood pressure window on the horizontal part of the ΔPAT/ΔPWV vs. blood pressure at left fingertip graph, to the right of the inflection point, which was 70 mmHg. A range of 75–95 mmHg was chosen based on published data on the lower limits of autoregulation in the brain and the kidneys (Carlström et al., 2015; Scheeren and Saugel, 2017). We performed the same analysis using different ranges (e.g., 80–100 mmHg) and observed the same results for the limits of autoregulation. Furthermore, if a particular range of distending blood pressures is within the limits of autoregulation, the specific range should have a slope close to 0. The slope of the regression analysis for the 75–95 mmHg range was 0.097 (*p* = 0.69) and for the 80–100 mmHg range 0.25 (*p* = 0.36). As blood pressure at the tip of finger increased above 112 mmHg, we saw changes in both ΔPAT and ΔPWV compared to those at a known autoregulation range of 75-95 mmHg, which is consistent with the theory that the vasculature operates on distending pressures above the upper limit of autoregulation. Using our noninvasive approach, we were able to establish PWV based limits of peripheral artery autoregulation (56–112 mmHg) in human peripheral arteries of the upper extremity. Intriguingly they are very close to the known blood flow based limits of autoregulation described for the brain and kidneys. Thus, the peripheral arteries of the arm could potentially be used as a window into non-invasive assessment of responses in the arterial system to changes in blood pressure and establishing at least the lower limit of autoregulation in individual subjects.

From our data, it is evident that a low distending pressure results in a profound decrease in peripheral arteries PWV in healthy individuals. Interestingly, a notable change in the slope of both ΔPAT and ΔPWV over the distending blood pressure occurs at 70 mmHg. We did not observe such a notable change in the slope at high blood pressures, likely as lowering the arm cannot result in pressures high enough to overcome the upper limit of autoregulation in all subjects.

In an elegant study using ultrasonographic and Doppler techniques, Eiken and Kölegård, showed that indeed changes in intravascular pressure in the range of 75–275 mmHg lead to significant changes in arterial stiffness of peripheral arteries as evident by changes in flow and diameter (Eiken and Kölegård, 2004). Notably, they found that there were greater changes in the flow of the arm arteries compared to the arteries of the leg for the same changes in pressures above 160 mmHg. This indicates that the peripheral arterioles especially in the arm are sensitive to changes in distending pressure and present autoregulation behavior. The blood flow at the lowest pressure in their experiment (70 mmHg) did not change from the flow at 160 mmHg suggesting that they were not able to detect the lower limit of autoregulation in the experiments.

A study by Foo et al. examined pulse transit time (PTT) at different limb positions (Foo et al., 2005). They found that there were significant PTT changes in the limb that underwent positional change, relative to a baseline control value. When the limb was vertically raised, PTT increased; this is consistent with our data. The authors attributed this increase in PTT to changes in hydrostatic pressure and regulation mechanisms within the limbs. They concluded that PTT is indicative of local circulatory responses, suggesting the existence of an autoregulatory mechanism within the arteries of the arm.

In our previous study, we showed that PAT changes significantly depend on the site of measurement (ear lobe, index finger, or big toe) (Obata et al., 2017). In that study, the volunteers changed their positions from supine to sitting to standing. Changing position from supine to standing introduces hydrostatic pressure gradients in the thoracic, abdominal, and peripheral arteries of the lower extremities. However, the observed changes in PAT and PWV did not allow to distinguish hydrostatic effects between the central and peripheral circulation. Moreover, changing the entire body position from supine to standing introduces significant global hemodynamic effects (such as heart rate and blood pressure changes) which affected arterial wall tension and PWV independent of hydrostatic blood pressure changes. This did not allow us to distinguish hydrostatic blood pressure changes from hemodynamically induced changes in the arterial wall.

The current study design enabled us to assess the effect of hydrostatic blood pressure changes specifically on peripheral arteries and to minimize the global changes in the hemodynamic state. Changing the position of only one arm introduced minimal hemodynamic effects compared to postural changes of the whole body. The right arm was consistently kept at the level of the heart to serve as an intrinsic control, canceling out any potential systemic hemodynamic effects. Thus, we quantified the relationship between distending pressure (MAP plus hydrostatic pressure changes due to changes in arm position) and ΔPAT with ΔPWV. Importantly, it allowed us to calculate the limits of autoregulation in a peripheral artery in a simple and non-invasive way. It would be intriguing to see if there is a correlation between limits of autoregulation in a peripheral artery and the brain.

### Limitations

Our study has several limitations. For MAP, we used only one single baseline blood pressure reading at position A0. There is a possibility that MAP would change with changes in the arm position. However, we believed that those changes will be minimal. Inflating the blood pressure cuff itself will induce local changes by squeezing the arm arteries, which we wanted to avoid. We used the peak of the R-wave on the ECG as the reference point for PAT and substituted pulse transit time with PAT for PWV calculations. The pre-ejection phase in healthy subjects is about 35 ms (Biering-Sørensen et al., 2016), whereas the PAT is in range of 150–300 ms, thus PAT overestimates true pulse transit time by 15%. However, we calculated ΔPAT between the left and right arm, which would cancel out the pre-ejection time and the time pulse wave spend in central aorta. To determine the limits of autoregulation, we used each position data point for every subject; this means, however, that there were a limited number of data points for each subject. Data points at angle increments for each subject would be more adequate in order to generate a smoother and more comprehensive model, which would yield more precise limits of autoregulation in individual subjects. Finally, the limits of autoregulation in our experiments are based on PWV instead of flow velocity. Without simultaneous measurements of Doppler flow velocity we cannot guarantee that PWV based limits of autoregulation are same as flow velocity based.

## Conclusions

In conclusion, our study shows that changes in hydrostatic pressure due to positional changes of the arm significantly affects peripheral arterial stiffness. This allows us to estimate the limits of regional autoregulation based on PWV.

## Author contributions

VB conceived the study and developed the protocol; AT, YO, NA, and VB collected data; AT, YO, PR, NA, and VB analyzed and interpreted the data; AT, YO, PR, DB, JS, and VB drafted and revised the manuscript. All authors read and approved the final version of the manuscript.

### Conflict of interest statement

The authors declare that the research was conducted in the absence of any commercial or financial relationships that could be construed as a potential conflict of interest.
