# Supplementary material for: A Pulse Wave Velocity Based Method to Assess the Mean Arterial Blood Pressure Limits of Autoregulation in Peripheral Arteries
Source: Front Physiol. 2017 Nov 2;8:855. doi: 10.3389/fphys.2017.00855 (PMC5681752; doi:10.3389/fphys.2017.00855)
Supplement: Supplementary file 1 [file Image1.PDF]

Supplementary Material

A Pulse Wave Velocity Based Method to Assess the Mean Arterial Blood Pressure Limits of Autoregulation in Peripheral Arteries

Ananya Tripathi, Yurie Obata, Pavel Ruzankin, Narwan Askaryar, Dan E. Berkowitz, Jochen Steppan, Viachaslau Barodka*

*** Correspondence:** Viachaslau Barodka: vbarodk1@jhmi.edu

Supplementary Figures

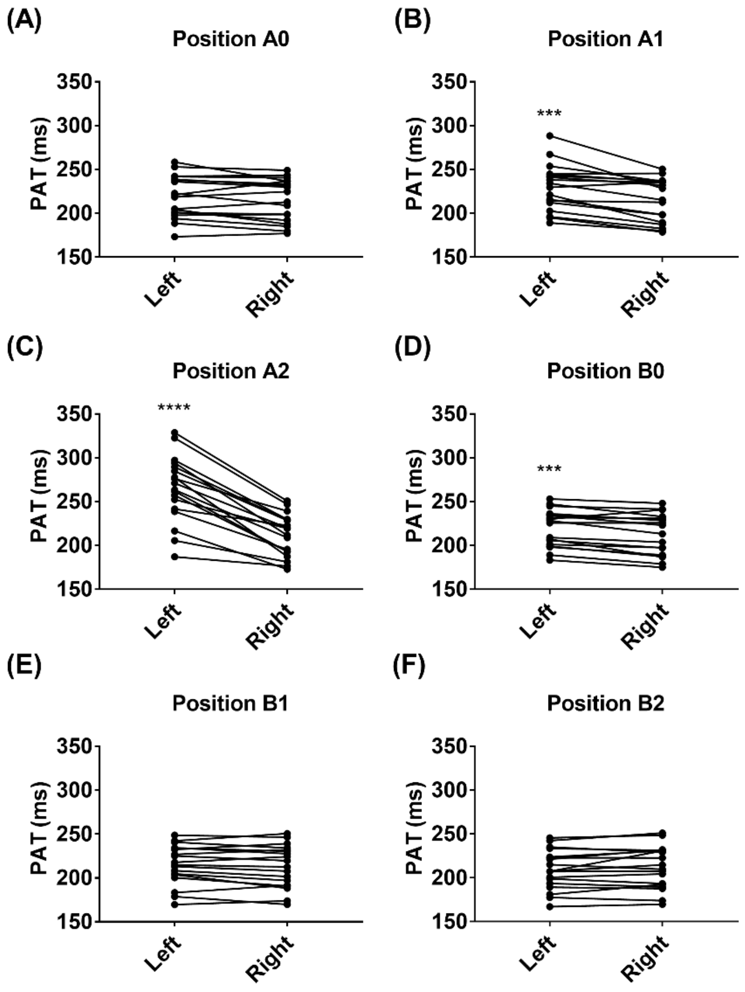

Supplementary Figure 1. PAT comparisons.
Left PAT vs. right PAT for individual subjects at each position. The right arm was consistently in the horizontal position while the left arm changed position. (A) Comparison of left PAT and right PAT at

position A0. (B) Comparison of left PAT and right PAT at position A1. (C) Comparison of left PAT and right PAT at position A2. (D) Comparison of left PAT and right PAT at position B0. (E) Comparison of left PAT and right PAT at position B1. (F) Comparison of left PAT and right PAT at position B2.
PAT: pulse arrival time; position A0: baseline horizontal position; position A1: left forearm extended vertically towards ceiling (half-up); position A2: left forearm and upper-arm extended vertically towards ceiling (up); position B0: horizontal position in the middle of the study; position B1: left forearm extended vertically towards floor (half-down); position B2: left forearm and upper-arm extended vertically towards floor (down); ms: milliseconds. ****: $p < 0.0001$; ***: $p < 0.001$.

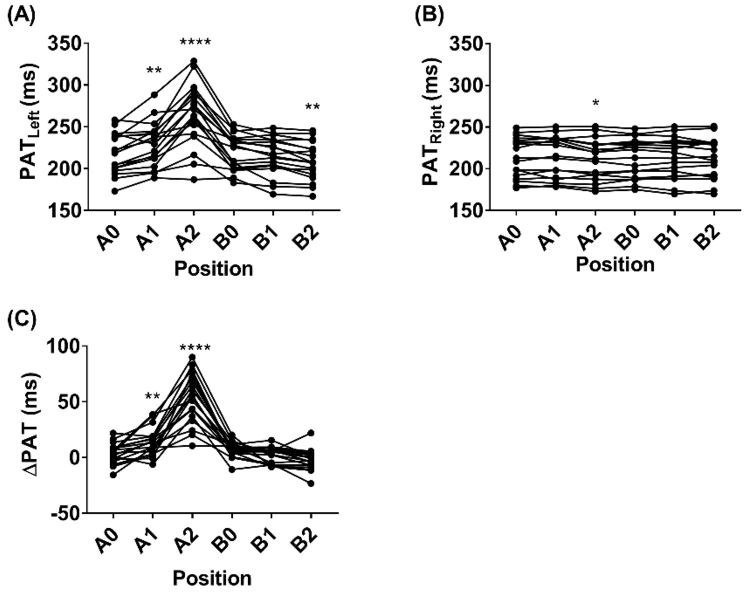

Supplementary Figure 2. PAT for all positions.
PAT and ΔPAT for individual subjects at all positions during the trial. (A) Left PAT at positions A0, A1, A2, B0, B1, A2. (B) Right PAT at positions A0, A1, A2, B0, B1, B2. (C) ΔPAT at positions A0, A1, A2, B0, B1, B2.
PAT: pulse arrival time; ΔPAT: difference between the pulse arrival time at the left index finger and right index finger; position A0: baseline horizontal control position; position A1: left forearm extended vertically towards ceiling (half-up); position A2: left forearm and upper-arm extended vertically towards ceiling (up); position B0: horizontal position in the middle of the study; position B1: left forearm extended vertically towards floor (half-down); position B2: left forearm and upper-arm extended vertically towards floor (down); ms: milliseconds. ****: $p < 0.0001$; **: $p < 0.01$; *: $p < 0.05$.

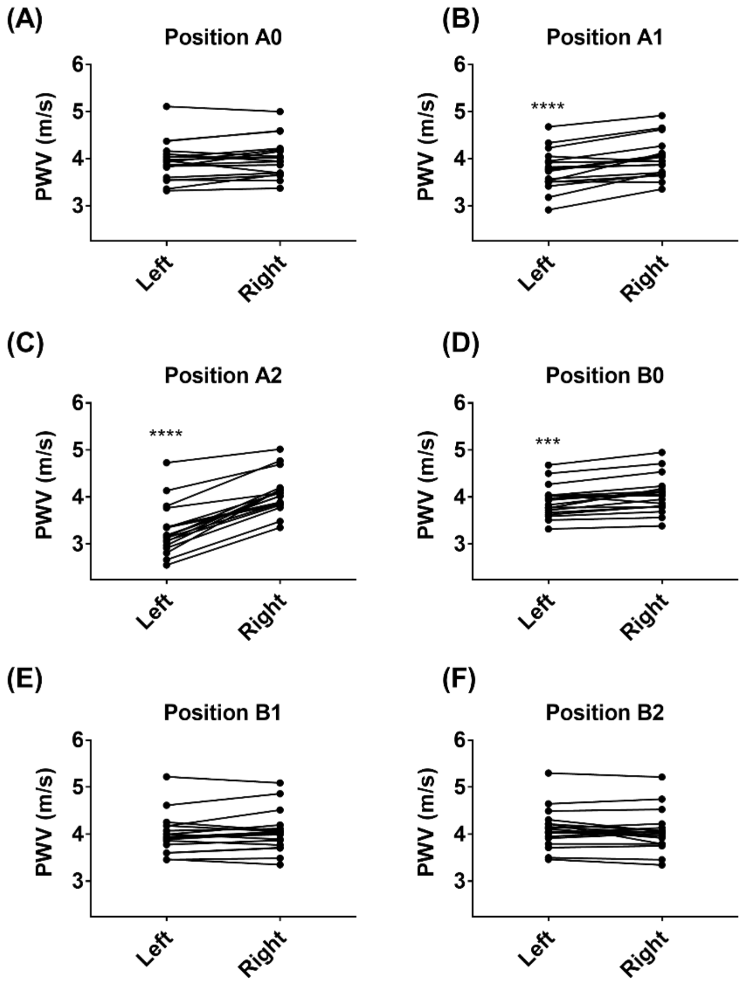

Supplementary Figure 3. PWV comparisons.

Left PWV vs. right PWV for individual subjects at each position. The right arm was consistently in the horizontal position while the left arm changed position. (A) Comparison of left PWV and right PWV at position A0. (B) Comparison of left PWV and right PWV at position A1. (C) Comparison of left PWV and right PWV at position A2. (D) Comparison of left PWV and right PWV at position B0. (E) Comparison of left PWV and right PWV at position B1. (F) Comparison of left PWV and right PWV at position B2.

PWV: pulse wave velocity; ΔPAT: difference between the pulse arrival time at the left index finger and right index finger; position A0: baseline horizontal control position; position A1: left forearm extended vertically towards ceiling (half-up); position A2: left forearm and upper-arm extended vertically towards ceiling (up); position B0: horizontal position in the middle of the study; position B1: left forearm extended vertically towards floor (half-down); position B2: left forearm and upper-arm extended vertically towards floor (down); m/s: meters per second. ****: $p < 0.0001$; ***: $p < 0.001$.

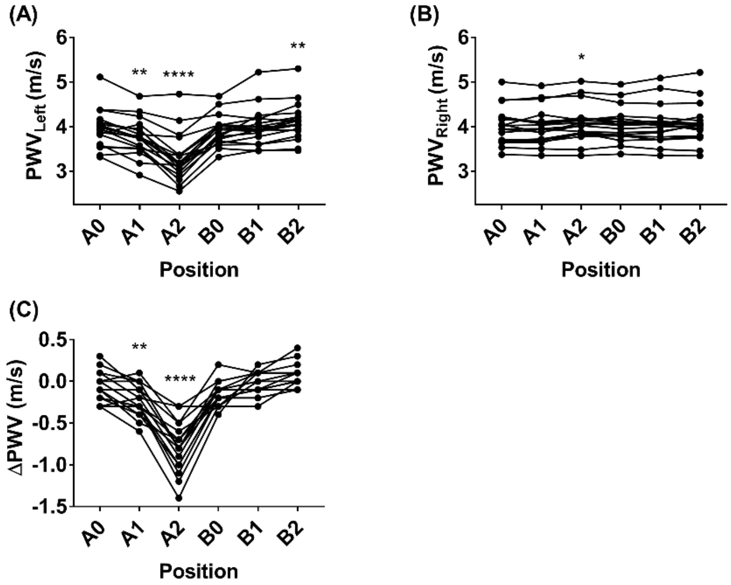

Supplementary Figure 4. PWV values for all positions.
PWV for individual subjects at all positions. (A) Left PWV at positions A0, A1, A2, B0, B1, and B2. (B) Right PWV at positions A0, A1, A2, B0, B1, and B2. (C) ΔPWV at positions A0, A1, A2, B0, B1, and B2.
PWV: pulse wave velocity; ΔPWV: difference between the pulse wave velocity at the left index finger and right index finger; position A0: baseline horizontal control position; position A1: left forearm extended vertically towards ceiling (half-up); position A2: left forearm and upper-arm extended vertically towards ceiling (up); position B0: horizontal position in the middle of the study; position B1: left forearm extended vertically towards floor (half-down); position B2: left forearm and upper-arm extended vertically towards floor (down); m/s: meters per second. ****: $p < 0.0001$; **: $p < 0.01$; *: $p < 0.05$.
